# A Developmental Role of the Cystic Fibrosis Transmembrane Conductance Regulator in Cystic Fibrosis Lung Disease Pathogenesis

**DOI:** 10.3389/fcell.2021.742891

**Published:** 2021-10-11

**Authors:** Elena N. Huang, Henry Quach, Jin-A Lee, Joshua Dierolf, Theo J. Moraes, Amy P. Wong

**Affiliations:** ^1^Program in Developmental and Stem Cell Biology, Hospital for Sick Children, Toronto, ON, Canada; ^2^Department of Laboratory Medicine & Pathobiology, University of Toronto, Toronto, ON, Canada; ^3^Program in Translational Medicine, Hospital for Sick Children, Toronto, ON, Canada

**Keywords:** stem cells, disease modeling, lung development and pulmonary diseases, Wnt/β-catenin, cystic fibrosis

## Abstract

The cystic fibrosis (CF) transmembrane conductance regulator (CFTR) protein is a cAMP-activated anion channel that is critical for regulating fluid and ion transport across the epithelium. This process is disrupted in CF epithelia, and patients harbouring CF-causing mutations experience reduced lung function as a result, associated with the increased rate of mortality. Much progress has been made in CF research leading to treatments that improve CFTR function, including small molecule modulators. However, clinical outcomes are not necessarily mutation-specific as individuals harboring the same genetic mutation may present with varying disease manifestations and responses to therapy. This suggests that the CFTR protein may have alternative functions that remain under-appreciated and yet can impact disease. In this mini review, we highlight some notable research implicating an important role of CFTR protein during early lung development and how mutant CFTR proteins may impact CF airway disease pathogenesis. We also discuss recent novel cell and animal models that can now be used to identify a developmental cause of CF lung disease.

## Introduction

**Cystic fibrosis** (CF) is a lethal, monogenic disorder involving autosomal recessive mutations of the CF transmembrane conductance regulator (*CFTR*) gene encoding the CFTR protein. CFTR functions as an anion channel that regulates fluid transport across specialized epithelia. Loss of CFTR channel expression or function due to disease-causing mutations leads to defects in ion and fluid transport into the airway lumen (Bergeron and Cantin, 2019) which ultimately lead to persistent microbial presence causing chronic inflammation and irreversible lung damage (Ratjen et al., 2015). To date, there have been over 2,000 CFTR mutations identified, of which over 360 are considered disease-causing mutations^1^. Disease-causing CF mutations are classified into seven classes that contribute to disease severity (De Boeck, 2020). Class I protein production mutations produce defective mRNA that interfere with the production of the full-length mutant protein. The most common Class II (i.e., F508del) mutations are protein processing mutations that interfere with the polypeptide folding. Class III (i.e., G551D) are gating mutations that impair CFTR channel opening while Class IV and V mutations are conduction and insufficient production mutations that impair anion conduction or reduce protein expression on the surface of the cells, respectively. Finally, Class VI and VII affect surface protein stability or no expression of mRNA. CF is further complicated by phenotypic variances in patients even with the same genetic mutation and this may be a result of disease-causing gene modifiers (Strug et al., 2016) and environmental factors (Ramsay et al., 2016). Novel drugs are improving mutant CFTR protein function, however many challenges still remain. There are considerable variations in drug responses and current drugs are not effective for all CF mutations (Laselva et al., 2021). Multiple drug therapies are available for patients such as Kalydeco^TM^ for patients >4-months with at least one G551D mutant allele, and the latest life-saving drug Trikafta^TM^ approved for the majority of CF individuals with at least one F508del mutant allele in patients >6 years in the US and >12 years in Canada. Young children present with similar disease pathologies (Waters and Ratjen, 2015; Ramsey et al., 2017) to adults and delaying therapy may have negative implications for disease progression and severity. CF disease pathogenesis during development is not completely understood and requires additional investigation using relevant models for which simple knockout models (both animal and cell lines) cannot capture. This mini review aims to highlight evidence of early disease manifestations in the developing lung and the need to revisit the role of CFTR in fetal lung development, especially in the advent of more recent animal and relevant cell models which will provide insight into CFTR mutations and its impact on lung maturation and function.

**The human lung** emerges from the ventral foregut endoderm (Cardoso and Lü, 2006). Lung development is categorized into five distinct phases: embryonic, pseudoglandular, canalicular, saccular, and alveolar. The lung buds emerge in the embryonic phase. In the pseudoglandular phase, the primitive lung buds undergo branching morphogenesis coordinated by the crosstalk between the developing epithelial and surrounding mesenchymal cells. Early vessels emerge simultaneously to form the pulmonary vasculature. Differentiation of several lung cell types begins and is regulated through proximal-distal patterning (Lu et al., 2001). In the canalicular phase, further differentiation leads to formation of specialized epithelial cell subtypes and the appearance of the primitive alveolar cells. Extensive growth of the fetal lung with expansion of the air sacs, thinning of connective tissue, deposition of extracellular matrices, and the formation of surfactant proteins marks the saccular phase. Finally, secondary septations in the alveolar phase form complex alveoli that optimize gas exchange with a concomitant increase in surfactant production. The human lung continues to develop well past childhood years (Schittny, 2017), and by adulthood, it will consist of >60 cell types (Travaglini et al., 2020) that collectively play a vital role in breathing, gas exchange, acid-base balance, metabolism, and immunity. At this point, CFTR expression in the adult airways is restricted to the apical membrane of certain specialized cell types such as ciliated and alveolar epithelial cells (Regnier et al., 2008), secretory cells and submucosal glands (Engelhardt et al., 1992), and ionocytes (Montoro et al., 2018).

**Spatio-temporal expression of CFTR in the developing lungs** suggests it may play an important role in organ formation. High levels of *CFTR* mRNA are found in the developing pancreas, kidneys, liver, and gut (Amaral et al., 2020). CFTR protein is also broadly expressed in the developing human fetal airways with a progressive increase in expression by mid-gestation (∼75-fold higher than adult lung) (Marcorelles et al., 2007). Detectable as early as the pseudoglandular stage around 7 weeks, CFTR is found diffusely in the cytoplasm of multipotent progenitor cells (Trezise et al., 1993). After peaking at mid-gestation, CFTR expression declines, remains low and expressed on the apical membrane of a subset of bronchial epithelial cells (Trezise et al., 1993; Marcorelles et al., 2007; Saint-Criq and Gray, 2017). Interestingly, the decline in CFTR expression correlates with cellular differentiation and lineage commitment. A comparison of CFTR expression in lungs of healthy and CF fetuses showed a 3-week delay in CFTR expression in the latter group until 15 weeks gestation (Marcorelles et al., 2007). The morphogenetic impact of this delay remains unknown but evidence of congenital defects as a result of mutant CFTR and some proposed mechanisms of how CFTR may regulate developmental processes are suggested.

**Lung malformations and proinflammatory changes as a result of CFTR disruption** have been found in fetal and newborn lungs. In rodents, malformations of the tracheal cartilaginous rings manifest in CFTR-deficient mice reduced breathing rate, suggestive of a role for CFTR in tracheal development (Bonvin et al., 2008). Moreover, *in utero* knockdown of CFTR in rats displayed classic CF morphologies of chronic inflammation, airway fibrosis and reactive airway disease (Cohen and Larson, 2005). In humans, abnormalities of the respiratory system caused by CF disease are observed in early childhood (Larson and Cohen, 2005). Studies of CF fetal lung tissue have demonstrated morphological abnormalities in tight junctions, dysfunction and absence of cilia in tracheal cells, and tracheal atrophy (Ornoy et al., 1987; Castellani et al., 2017). A study in newborn CF children found decreased tracheal lumen size (Meyerholz et al., 2010a). Although most CF infants are asymptomatic, 80.7% demonstrate abnormal lung changes and 77.2% exhibit detectable lung inflammation (Sly et al., 2009). Indeed, intensive CF early surveillance programs such as the Australian Respiratory Early Surveillance Team for Cystic Fibrosis (ARESTCF) have shown that early lung disease in infants and young children can lead to irreversible lung damage. Therefore, optimal CF management may require early treatment but the potential risks of treatment-related side effects to the developing lung which remain unknown.

***In utero* correction of CFTR function can impact lung development and function**. *In utero* delivery of CFTR has previously been shown to improve pulmonary under-development in a nitrofen-induced rat model of pulmonary hypoplasia (Larson and Cohen, 2006). Similarly, Sun et al. (2019) showed that a one-time antenatal administration of CFTR modulator rescued pancreatic and intestinal functions, reduced airway mucus production, and improved postnatal growth and survival of CF ferret kits. However, withdrawal of treatment at any point after birth reversed the phenotype resulting in bacterial infections and lung exacerbations similar to untreated animals. Together, both studies implicate CFTR in regulating lung morphogenesis and can impact postnatal lung function. Therefore, correcting prenatal defects may have a beneficial impact on reducing CF disease pathogenesis as has been suggested in the ARESTCF studies, and improve therapeutic outcomes for young CF children.

**CFTR regulates fluid distension and mechanical stimulation** of the developing lung. CFTR activation in the hypercalcemic fetal lung is facilitated by an extracellular calcium-sensing receptor (CaSR) that acts through activation of adenylate cyclase (Brennan et al., 2016). This secretion occurs during pseudoglandular branching and budding of airways and provides the lung with the distending pressure necessary for the expansion of the lungs. *In utero* CFTR gene transfer significantly affects the expression of proteins involved in smooth muscle contraction through the PI3 kinase and phospholipase-C pathways, modulating cytoskeletal tension and stretch induced differentiation of fetal lungs in a Rho kinase-independent manner (Cohen and Larson, 2006). In CF pig models, defects in CFTR-dependent anion transport and corresponding fluid secretion led to bud hypo-distension in the pseudoglandular airways that were unresponsive to CFTR agonists, forskolin/IBMX stimulation (Meyerholz et al., 2018). Altogether, these studies points to a mechanical role of CFTR in regulating growth of the developing fetal lung.

**A putative role of CFTR in regulating epithelial differentiation** has been previously shown (Brezillon et al., 1995; Dupuit et al., 1995). Mutant CFTR (F508del) localizes to the apical membrane of cells to a lesser degree (Kälin et al., 1999; Penque et al., 2000), suggesting that abnormal CFTR localization may be dependent on the differentiation state of the cell. In mice, *in utero* over-expression of CFTR increases bronchial cell differentiation and proliferation at the expense of alveolar development (Larson et al., 2000), resulting in death. In a rat model, transient *in utero* knockdown of CFTR altered alveolar Type II cell phenotype and associated surfactant homeostasis (Gad et al., 2009). An increase in CFTR mRNA in nasal epithelial cells has been shown to correlate with an increase in mucin and aquaporin expression (Jun et al., 2001). It has also been observed that genes associated with cilia biogenesis are down-regulated in F508del epithelia when compared to non-CF (Clarke et al., 2013). Overall, these studies implicate a role of CFTR in cellular differentiation. However, the mechanism(s) of how CFTR regulates differentiation during development is unclear.

**FIGURE 1:**
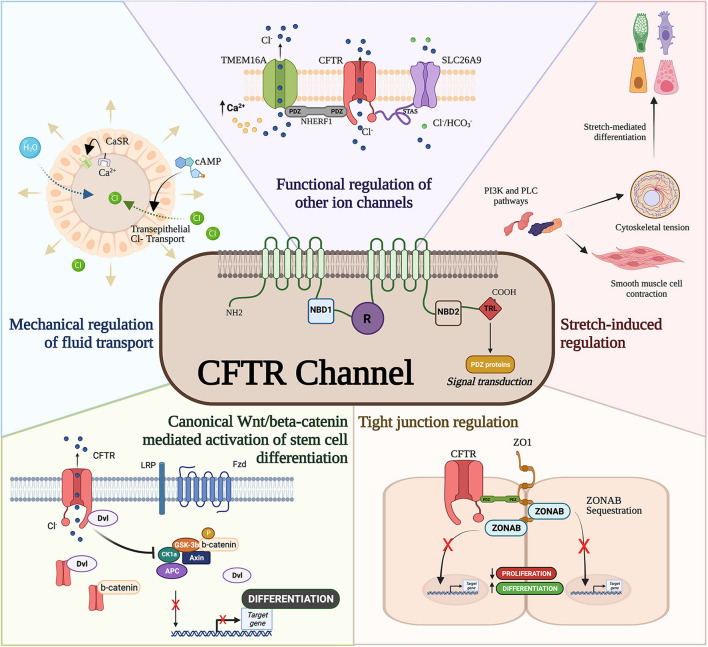
Cystic fibrosis transmembrane conductance regulator (CFTR) developmental interactome. The CFTR channel has been associated with developmental processes and regulatory pathways. CFTR mediates mechanical forces for airway development, functionally regulates other ion channels, drives differentiation through ZO-1, interferes with canonical Wnt/β-catenin activation of stem cell differentiation, and modulates smooth muscle cell contraction and cytoskeletal tension.

Building evidence supports a direct regulatory role of CFTR protein in interacting with PDZ-containing signal transducers such as β-catenin (Liu et al., 2017), binding to cytoskeletal proteins such as zonula occludens-1 (ZO)-1, and regulating other ion channels (Benedetto et al., 2017; Figure 1) which can affect epithelial differentiation and proliferation. The canonical Wnt/β-catenin signaling plays a crucial role in normal lung development by regulating cellular differentiation and proliferation of uncommitted progenitor cells (Cardoso, 2001; De Langhe and Reynolds, 2008). Canonical Wnt activation through binding of Wnt ligands (i.e., Wnt3a) to its receptor Frizzled activates a cascade of signal transduction via the disheveled protein. This leads to the disruption of the Axin/APC/GSKβ degradation complex and in turn stabilizes β-catenin. Nuclear translocation of β-catenin binds to TCF/LEF transcription factors to transactivate Wnt target genes that regulate cellular proliferation and differentiation (MacDonald et al., 2009). An inverse relationship exists between CFTR and β-catenin activation as antisense-mediated knockdown of CFTR expression in the developing mouse lungs prolonged Wnt activity (Cohen et al., 2008). Overexpression of CFTR during lung development shortened Wnt activity and increased bronchial cell differentiation. Deep proteomic analysis of CF and wild-type cells revealed an association between wild-type CFTR with Wnt signaling components that was lost in mutant (F508del) CFTR (Pankow et al., 2015), further strengthening the observation of a direct relationship between CFTR and Wnt activity. Interestingly, CFTR/β-catenin interplay has also been demonstrated in other models such as kidney development (Zhang et al., 2017), intestinal inflammation (Liu et al., 2016), and pancreas development (Hyde et al., 1997).

The last three amino acids in the C-terminal domain of the CFTR protein contain a PDZ domain required for apical membrane polarization (Moyer et al., 1999). While apical localization of CFTR is important for epithelial differentiation (Hollande et al., 1998; Castillon et al., 2002) and suppression of epithelial-mesenchymal transition (Corvol et al., 2018), CFTR’s PDZ binding domain is known to directly and indirectly regulate developmental pathways. Direct binding of CFTR to ZO-1 sequesters the ZO-1 associated nucleic acid binding (ZONAB) protein in tight junctions obstructing ZONAB nuclear translocation where it can activate genes associated with proliferation and differentiation (Ruan et al., 2014). CFTR/ZO-1 interaction has also been shown to form a complex with ezrin to induce ciliogenesis and secretory cell differentiation in dedifferentiated airway cells (Castillon et al., 2002). Furthermore, F508del CFTR mutations are capable of inhibiting intracellular transport activity of the SLC26A9 ion channel (Bertrand et al., 2017) through PDZ binding domains to the scaffolding protein, Na^+^/H^+^ exchanger regulatory factor isoform 1 (NHERF1). Interestingly, CFTR-NHERF1 interactions has been also shown to regulate Transmembrane member 16A (TMEM16A) (Benedetto et al., 2017), a calcium activated chloride channel previously shown to regulate fetal airway epithelial differentiation (He et al., 2020). Therefore, whether directly or indirectly, CFTR appears to be important in epithelial differentiation.

**FIGURE 2:**
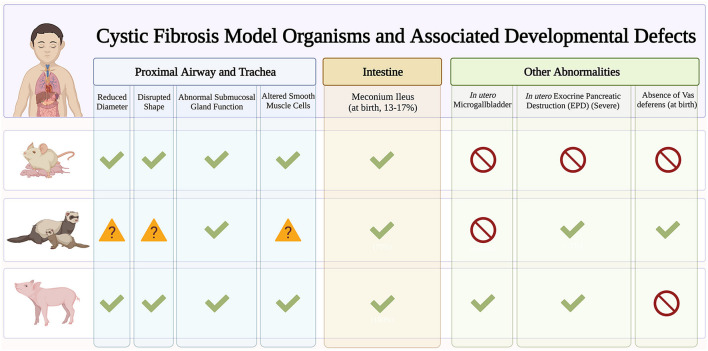
Early developmental pathology in cystic fibrosis (CF) animal models. Early manifestations of CF disease are recapitulated in different CF animal models. Checkmarks indicate presence of symptoms. Absence of symptoms is indicated by the red circle symbol. The question mark symbols indicate further investigation needed.

**New animal and advanced cell models** have emerged to elucidate the role of CFTR in lung development and cellular differentiation. This will provide important clues into why CF infants exhibit pulmonary abnormalities such as airway inflammation (Armstrong et al., 1997; Verhaeghe et al., 2007), functional (Beardsmore et al., 1988) and structural (Long et al., 2004) abnormalities, tracheal epithelial atrophy, and absence of cilia (Ornoy et al., 1987), and increased susceptibility to lower respiratory tract infections (Hiatt et al., 1999; Deschamp et al., 2019). While mouse models have provided important insight into the developmental pathways driving lung morphogenesis (Morrisey and Hogan, 2010), CF knockout mouse models do not recapitulate lung pathologies seen in humans (Guilbault et al., 2007). Here we focus on two animal models, ferrets and pigs, that do develop similar lung CF pathologies and highlight the emerging use of induced pluripotent stem cell (iPSC) in modeling airway development (Figure 2).

The ferret CF model exhibits multiorgan pathologies similar to human disease. These include pancreatic insufficiency, intestinal obstruction, stunted growth, and increased susceptibility to lung infections (Yan et al., 2015). As such, ferrets are great models to understand basic CF epithelial electrophysiology (Fisher et al., 2013) and assess small molecule and gene-based therapies (Cmielewski et al., 2014; Sun et al., 2019). Knockout ferrets develop severe lung and intestinal disease within the first week of life (Sun et al., 2010) leading to significant mortality soon after birth (Sun et al., 2014). Emerging ferret models with human mutations such as the G551D are useful for studying biological problems in a mutation-dependent manner (Semaniakou et al., 2019). With a short (42 day) gestational period, ferrets are great models to characterize the effects of modulator treatment *in utero*. In fact, *in utero* treatment with CFTR modulators improve postnatal CF kit lung functions which suggests fetal CFTR function is important in limiting pathogenic changes affecting postnatal function.

Pig models are suitable for studying lung development and diseases since they share many anatomical features with humans. CF pigs harboring F508del mutations have been generated that exhibit the same disease phenotype as humans (Rogers et al., 2008; Ostedgaard et al., 2011). CF piglets exhibit tracheal malformations similar to those seen in CF infants, including reduced lumen diameter and circularity (Meyerholz et al., 2010a, 2018) that contribute to abnormal particle distribution (Awadalla et al., 2014). CF piglets also exhibit no signs of inflammation at birth, but quickly develop lung disease and infections, resembling human disease (Stoltz et al., 2010). Human pancreatic disease such as small exocrine pancreas and inflammation are also observed in CF piglets. Interestingly, CF pancreatic disease begins *in utero* with activation of inflammatory and remodeling pathways (Abu-El-Haija et al., 2012). However, one important caveat is that CF pigs are prone to meconium ileus that often necessitates euthanasia despite surgical efforts (Meyerholz et al., 2010b; Guillon et al., 2015), and this may preclude the use of CF pigs in studying the long-term effects of CFTR in development.

Regulatory concerns over the use of human fetal tissues for research and the scarcity of fetal tissues with rare congenital disorders, is a major obstacle to understanding human lung development. Therefore, iPSC has become an attractive surrogate for fundamental discoveries in human development. These cells are artificially reprogrammed cells from somatic cells that phenocopy and function like embryonic stem cells (Takahashi and Yamanaka, 2006). These iPSC offer several advantages over cell lines and primary epithelial cells: (1) iPSC are an unlimited source of patient-derived cells that harbor the patient genotype allowing the study of patient-specific disease and therapies, (2) iPSC harboring various classes of CFTR mutations can be used to study mutation-dependent effects, (3) iPSC differentiation models stepwise developmental changes and therefore differentiation to fetal phenotypes offer an incredible opportunity to study mechanisms of development, (4) iPSC from one individual can generate multiple tissue/cell types and therefore offer a great source of cells to understand disease in tissue-specific context.

Development of differentiation protocols to generate lung cells have been established (Wong et al., 2012, 2015; Firth et al., 2014; Huang et al., 2014; Konishi et al., 2016; McCauley et al., 2017; Leibel et al., 2020). The differentiation process involves precise temporal and dose-dependent exposure of the cells to specific recombinant proteins and/or small molecules affecting key signaling pathways. Generally, the first major differentiation steps involve generation of definitive endoderm cells, followed by patterning to form the anterior ventral foregut endoderm that will form the earliest lung cells. Further differentiation generates fetal lung progenitors and requires key morphogens such as fibroblast growth factors, bone morphogenetic proteins and Wnts. Finally, exposure to air *in vitro* using air liquid interface cultures to mimic the microenvironment of the postnatal lung induces maturation and polarization of the epithelia. Recent sequencing technologies have mapped the faithful lung lineage differentiation of iPSC in modeling development compared to their *in vivo* tissue counterparts (Miller et al., 2019; Hurley et al., 2020; Hawkins et al., 2021). It has also been shown that iPSC-derived fetal lung cells transcriptionally match late pseudoglandular fetal lung epithelial cells (Ngan et al., 2021). Human iPSC-derived fetal lung cells provide an opportunity to determine the role of CFTR in pseudoglandular lung epithelial development. With iPSC harboring different CFTR mutations, the effects of CFTR mutations in lung epithelial lineage development and the long-term effects of modulator therapy in epithelial development can be studied. Overall, *in vitro* hPSC differentiations are powerful complementary models to study developmental mechanisms underlying genetic diseases.

## Summary

Investigating the pleiotropic roles of CFTR during development and the impact of defective CFTR *in utero* is critical to understanding CF disease pathogenesis. Recent advances in cell and animal models will improve our understanding of an “old disease” from a developmental lens and potentially lead to the identification of novel therapies aimed at reversing or limiting disease progression.

## Author Contributions

EH and HQ: conceptualization, methodology, validation, formal analysis, investigation, writing—original draft, writing—review and editing, and visualization. J-AL, JD, and TM: writing—review and editing. AW: resources, writing—review and editing, funding acquisition, and supervision. All authors contributed to the article and approved the submitted version.

## Conflict of Interest

The authors declare that the research was conducted in the absence of any commercial or financial relationships that could be construed as a potential conflict of interest.

## Publisher’s Note

All claims expressed in this article are solely those of the authors and do not necessarily represent those of their affiliated organizations, or those of the publisher, the editors and the reviewers. Any product that may be evaluated in this article, or claim that may be made by its manufacturer, is not guaranteed or endorsed by the publisher.

## Footnotes

1
www.cftr2.org
